# The Relative Contributions of Live and Recorded Online Mindfulness Training Programs to Lower Stress in the Workplace: Longitudinal Observational Study

**DOI:** 10.2196/31935

**Published:** 2022-01-21

**Authors:** Ruth Q Wolever, Michael T M Finn, Denise Shields

**Affiliations:** 1 Osher Center for Integrative Health at Vanderbilt Department of Physical Medicine & Rehabilitation Vanderbilt University School of Medicine Nashville, TN United States; 2 Department of Pediatrics & Human Development Michigan State University Grand Rapids, MI United States; 3 eMindful Inc Orlando, FL United States

**Keywords:** mindfulness, live versus recorded, synchronous, asynchronous, workplace, digital health, online, live teaching, contribution, training, stress, longitudinal, observational, platform, eHealth, mental health

## Abstract

**Background:**

Despite numerous gaps in the literature, mindfulness training in the workplace is rapidly proliferating. Many “online” or “digital mindfulness” programs do not distinguish between live teaching and recorded or asynchronous sessions, yet differences in delivery mode (eg, face-to-face, online live, online self-guided, other) may explain outcomes.

**Objective:**

The aim of this study was to use existing data from an online mindfulness solutions company to assess the relative contribution of live and recorded mindfulness training to lower perceived stress in employees.

**Methods:**

Perceived stress and the amount of live and recorded online mindfulness training accessed by employees were assessed during eMindful’s One-Percent Challenge (OPC). The OPC is a 30-day program wherein participants are encouraged to spend 1% of their day (14 minutes) practicing mindfulness meditation on the platform. We used linear mixed-effects models to assess the relationship between stress reduction and usage of components of the eMindful platform (live teaching and recorded options) while controlling for potential reporting bias (completion) and sampling bias.

**Results:**

A total of 8341 participants from 44 companies registered for the OPC, with 7757 (93.00%) completing stress assessments prior to the OPC and 2360 (28.29%) completing the postassessment. Approximately one-quarter of the participants (28.86%, 2407/8341) completed both assessments. Most of the completers (2161/2407, 89.78%) engaged in the platform at least once. Among all participants (N=8341), 8.78% (n=707) accessed only recorded sessions and 33.78% (n=2818) participated only in the live programs. Most participants engaged in both live and recorded options, with those who used any recordings (2686/8341, 32.20%) tending to use them 3-4 times. Controlling for completer status, *any* participation with the eMindful OPC reduced stress (B=–0.32, 95% CI –0.35 to –0.30, SE=0.01, *t*_2393.25_=–24.99, *P*<.001, Cohen *d*=–1.02). Participation in live programs drove the decrease in stress (B=–0.03, SE=0.01, *t*_3258.61_=–3.03, *P*=.002, *d*=–0.11), whereas participation in recorded classes alone did not. Regular practice across the month led to a greater reduction in stress.

**Conclusions:**

Our findings are in stark contrast to the rapid evolution of online mindfulness training for the workplace. While the market is reproducing apps and recorded teaching at an unprecedented pace, our results demonstrate that live mindfulness programs with recorded or on-demand programs used to supplement live practices confer the strongest likelihood of achieving a significant decrease in stress levels.

## Introduction

As the practice of mindfulness continues to rise [1,2], there has been a concomitant emergence of online mindfulness programs [3]. Prior to the COVID-19 pandemic, an estimated 60% of mid-to-large–sized companies in the United States were offering mind-body programs such as mindfulness for their employees [4], with related services creating a billion-dollar industry [5]. The pandemic-related spike in stress and mental health issues [6,7] and the fact that such spikes impair multiple aspects of adult learning [8,9] have led to an even greater need to support employees [10,11]. As rapid growth of the online and workplace mindfulness industries has outpaced research, there is an increased demand for rigorous methodology to carefully evaluate workplace mindfulness training, particularly online training. Relevant literature has multiple confounds, including the fact that the relative impact of live (synchronous) and recorded (asynchronous) digital mindfulness training for the workplace is completely unknown.

Multiple systematic reviews have demonstrated solid evidence that mindfulness training has a positive impact on stress and other indicators of mental health [12-16]. In addition, mindfulness interventions clearly reduce anxiety and depression symptoms in a therapeutic context [17,18], and help even as a stand-alone intervention [19]. Mindfulness programs have consistently been shown to assist clinical patients, health care professionals, and multiple types of students, and further promise benefits for the workplace [20-22] by helping individuals to develop several skills [23]. For example, mindfulness meditation improves generalized attention, alerting, and multiple aspects of executive control [24,25]. There is a solid rationale for why mindfulness training may enhance workplace functioning [20]. Nonreactive attention on the present moment should enhance focus while lowering errors; practicing curiosity and new perspectives should invite creative problem-solving; and cultivation of the attitudes of mindfulness should enhance interpersonal relationships, customer service, and leadership [20]. Although little empirical work has actually assessed the potential improvement in occupational functioning [26], very recent meta-analyses on mindfulness interventions in the workplace show robust and consistent improvements on stress and indicators of well-being. One recent meta-analysis of 56 randomized controlled trials (RCTs) found that, compared to controls, *live* workplace mindfulness programs did effectively lower stress, mental distress, and burnout, while enhancing well-being, mindfulness, compassion, and job satisfaction (Hedges *g*=0.32-0.77). Importantly, improvements were sustained at short-term follow-up assessments up to 12 weeks [27]. Similarly, another recent meta-analysis of 35 RCTs found medium effects on stress, distress, anxiety, depression, and burnout, while also revealing small to medium effects for health, job performance, compassion/empathy, and well-being [21]. A third recent meta-analysis of 23 RCTs concurred in finding moderate effects for stress, distress, anxiety, and well-being [22]. Fortunately, this study further delved into the heterogeneity and rigor of the studies. Analyses of this heterogeneity and risk of bias clarified that findings for burnout, depression, and work performance measures are premature. Study method variability included differences in measures used, assessment timelines, selection and attrition bias, and limited use of active control groups. Intervention variability factors included differences in training content, dose (program hours, weeks), and delivery mode (eg, face to face, online live, online self-guided, other) [22]. Other researchers have also identified concerns with the state of the literature on mindfulness training in the workplace, noting its promise yet need to uplevel the rigor of studies to manage threats to internal and external validity [26]. For example, workplace-based mindfulness programs are typically adapted from training programs in other contexts, but do not generally utilize the same training structure, protocol, or time commitment as the original studies [22]. Although these adaptations may fit well in busy workplace settings, workplace programs are often assumed to be supported by evidence from the original studies while actually lacking empirical validation. Moreover, the literature on mindfulness at the worksite has largely relied upon cross-sectional studies rather than tracking longitudinal outcomes such as performance over time [28].

These literature shortcomings leave large gaps in understanding what moderates effective outcomes for mindfulness programs in the workplace. One significant moderator may well be program delivery venues. The same mindfulness training program will produce similar outcomes whether delivered in person or online, provided it is delivered live (ie, in real time or synchronous) [16]. Meta-analyses of 8 RCTs demonstrated a medium effect size (Hedges *g*=0.432) for perceived stress and a small effect size (Hedges *g*=0.275) for mindfulness measures in nonclinical populations in fully online, *live* mindfulness programs [29]. However, there may be a difference in the impact of workplace mindfulness programs when provided live (whether in person or online) versus fully recorded (“on demand” or “asynchronous”) or delivered through static apps. Only one review assessed delivery methods and found no moderating effects for program characteristics [27]. However, the authors acknowledged that their study may have been underpowered to detect program differences. In addition, their included studies had an unequal distribution of mindfulness training delivery venues (eg, on site, online). Although the unequal distribution allowed for greater external validity, it may have skewed the findings. The authors suggested that further studies are needed to explore the potential differential effects of mindfulness program characteristics.

“Online” or “digital mindfulness” studies frequently do not distinguish between live and recorded training. Furthermore, app-based, recorded web-based, or live online teaching [3,30] are often not differentiated, despite the dearth of evidence for all but live, online teaching. Researchers note the substantial amount of human and financial resources required to provide participants with live, tailored feedback versus automated feedback [29]. Given the relatively low cost and ease of reproducing automated feedback and on-demand content and apps, companies tend to “check the box” of providing mindfulness programs without empirical assessment of the program’s impact or the methodology of delivery. As a result, many “on-demand” programs and apps are being offered to employers and consumers, inappropriately leveraging empirical research conducted on live mindfulness training programs. For example, there are more than 2500 mindfulness-based apps available [31], yet rigorous efficacy research for such apps is meager [30,32,33]. In fact, in a 2015 review and evaluation of 560 mindfulness apps, there was only one efficacy study [33]. In the last 5 years, at least 34 RCTs have examined a mindfulness intervention where the main component was app-based mindfulness practice [34]. Although meta-analyses showed significant improvements in stress (Hedges *g*=0.46) using 15 of the trials, when one outlier trial was excluded, the benefit dropped by 30% (Hedges *g*=0.32 for 14 trials). The impact on anxiety, depression, and psychological well-being was also significant, albeit with small effect sizes (Hedges *g*=0.28-0.33). No significant effects were seen for distress or general well-being in the studies of mindfulness apps. More concerning were the methodological issues noted, including the fact that only 12 of the 34 trials had a low risk of bias in 5 or 6 potential bias domains. In general, the studies of apps lacked information on randomization and concealment allocation, and less than a third reported intention-to-treat analyses. It is thus unclear how effective recorded practices and apps are when not supplemented by live teaching.

Online mindfulness programs for the workplace come in many forms, ranging from live programs using fully validated protocols to workplace adaptations with no empirical backing to recorded programs and apps. Importantly, the relative impact of live online teaching versus recorded training is completely unknown. Given the rapid proliferation of digital mindfulness training opportunities to help participants acquire the skills necessary to negotiate today’s intense uncertainty and ongoing rise in stress, we wondered about the relative impact of participants’ use of recorded training materials versus live training. The objective of this study was to use existing data from a mindfulness solutions company to assess the contribution of live and recorded training to lower perceived stress.

## Methods

### Study Design and Ethics

Anonymized data were used to assess the immediate longitudinal impact of participating in a 30-day online mindfulness training platform. We evaluated the contributions of participant use of live programs and recorded offerings in explaining potential change in perceived stress. This nonhuman research study was provided an exemption by the Vanderbilt University Medical Center Institutional Review Board.

### Participants

Participants were unique users of eMindful Inc, who registered for the One Percent Challenge (OPC) between January 1, 2017, and February 29, 2020. Sociodemographic data on participants were obtained in two ways. Participants from the 2020 OPCs were asked to provide age, gender, and race/ethnicity in a preparticipation survey, but there was no requirement to do so. A second attempt at obtaining sociodemographic data was made by eMindful in culling the eligibility file data for earlier cohorts; data availability depended upon how the organization onboarded their employees with eMindful. In both cases, preferences of the individual organizations whose employees were participating were honored and data collection was often seen as a barrier to their employees getting started.

### Mindfulness Program

The OPC is a 30-day program wherein participants are encouraged to spend 1% of their day (14 minutes) practicing mindfulness meditation on the eMindful platform. Sixteen live, guided trainings are offered each workday by highly skilled mindfulness teachers with an average of 21 years of personal practice, 76% of whom have advanced degrees (eg, PhD, MD) or are licensed clinicians and 70% of whom are certified mindfulness teachers from the International Mindfulness Teacher Association. The live mindfulness sessions come in 3 formats, with an average attendance of 39.3 participants per session. Attendance at a given session has a wide range (9 to 183 participants); there is very high participation in the weekday morning sessions and very low attendance late nights and weekends. The most common format is the 14-minute Mindful Daily (topic), which consists of 3-4 minutes of didactic teaching on the application of mindfulness to a particular topic (eg, trouble falling sleep, coworker conflict, managing your “to do” list), followed by 10 minutes of guided mindfulness practice and a closing inquiry. Additional Mindful Daily (practice) sessions are 15 and 30 minutes in length and consist only of guided practice. Finally, there are multisession live programs that are related to chronic conditions. The multisession programs are delivered in sequenced, 55-minute sessions that aim to build skills in a particular order. They also combine didactic teaching and experiential practice, but allow more time for personal inquiry. While the focus of eMindul has been live teaching in a virtual classroom, the company developed an app with over 2500 recorded sessions that are “on demand” for participants to use to supplement or replace the live programs. The “on demand” options include high-quality reproductions of the live Mindful Daily sessions as well as recorded practice sequences designed specifically for common concerns (eg, Seven Days of Managing Workplace Stress, Return to the Workplace, Gratefully all in). Companies who provide the OPC to their employees typically use the OPC to introduce the new benefit to employees. Promotional material provided to companies to encourage participation includes emails, one-page flyers, posters, and brief newsletters focused on the relevance of mindfulness for employee well-being.

### Measures

#### Usage of the eMindful Platform

Backend data from eMindful provided two types of usage information: attendance (number of sessions attended, whether live or recorded) and days of mindfulness (number of days on which a participant practiced through use of the platform, no matter how many practices per day they completed).

#### Perceived Stress

Participants were asked to complete the Perceived Stress Scale-4 (PSS-4) [35] within 1 week of the start of the OPC and immediately post-OPC. Participants who did not respond postprogram were asked again through automated email and were allowed to take the PSS-4 up to 30 days after the closing of the OPC, regardless of the number of sessions completed. The PSS-4 is a 4-item abbreviated and validated version of the Perceived Stress Scale, which is a widely used measure of transactional stress that takes into consideration the balance between stressors and one’s perceived ability to manage them. The scale asks the following questions: In the last month, how often have you felt that you were unable to control the important things in your life? In the last month, how often have you felt confident about your ability to handle your personal problems? In the last month, how often have you felt that things were going your way? In the last month, how often have you felt difficulties were piling up so high that you could not overcome them?

Items are scored on a 0-4 Likert scale of how often they were experienced in the past month (Never to Very Often). Two of the items are then reverse-scored and item scores are summed to provide a total score. In addition to calculating pre and post perceived stress levels, an absolute difference was calculated for each individual to provide estimates of change over time.

### Statistical Plan and Data Presentation

Descriptive statistics were calculated on the sociodemographic, platform usage, and outcome data. We used linear mixed-effects models to assess the relationship between usage of the eMindful platform and reduction in stress. We were also interested in the relative impact of participation in live versus recorded sessions.

Given the practical nature of this real-world trial, and the level of potential nonrandom missingness, we first examined potential bias in completing both surveys on the basis of pre-OPC stress levels. This informed our inclusion of this factor in the modeling. We also had incomplete sociodemographic data that were assessed for potential sampling bias, and we examined the additional impact of relevant participant demographics on the model.

## Results

### Participants

A total of 8341 participants registered for the OPC. They were from 44 organizations who provided the OPC to their employees in the given timeline. Eight distinct OPC programs were run across the time period. The average number of participants per organization was 189.6 (SD 549.7), with a very wide range (1-3354 per organization). Thirty participants had a unique OPC that lasted 61 rather than 30 days, and hence were removed from the total count and analyses to avoid confounding the outcomes.

Participants who completed the preparticipation survey with sociodemographic data (age, gender, and race/ethnicity) or whose sociodemographic data were obtained from eligibility files allowed us to describe the sample. These two procedures produced information resulting in age being calculated for 68.04% (n=5675) of the total sample (N=8341), with a mean age of approximately 45 years, ranging from 20 to 79 years. Self-reported data on gender was available for 48.09% (n=4011) of the entire sample; the majority identified as female. Self-reported race/ethnicity data were available for 12.42% (n=1036) of the entire sample, with the majority identifying as white, followed by “nonwhite,” and preferring not to answer. Baseline demographics are presented in Table 1 organized by completer status.

**Table 1:** Demographics by completer status.

Characteristic			Did not complete both assessments (n=5934)		Completers of pre and post PSS-4^a^ (n=2407)		Total (N=8341)		*P* value	
**Age (years)**										<.001
	Responses, n	3509		2166		5675				
	Mean (SD)	44.3 (11.02)		46.3 (10.91)		45.1 (11.02)				
	Median (range)	44 (20-73)		47 (21-79)		45 (20-79)				
**Gender**										.09^b^
	Female, n (%)	1871 (81.24)		1413 (82.73)		3284 (81.87)				
	Male, n (%)	430 (18.67)		282 (16.51)		712 (17.75)				
	Prefer not to answer, n (%)	2 (0.10)		13 (0.76)		15 (0.37)				
	Missing, n	3631		699		4330				
**Race**										.67
	White, n (%)	132 (65.7)		560 (67.1)		692 (66.8)				
	Nonwhite, n (%)	56 (27.9)		220 (26.3)		276 (26.6)				
	Prefer not to answer, n (%)	13 (6.5)		55 (6.6)		68 (6.6)				
	Missing, n	5733		1572		7305				

^a^PSS-4: Perceived Stress Scale-4.

^b^Comparison based on male versus female.

### Data Availability and Statistics

#### Usage of the eMindful Platform

An estimated 65.93% (n=5499) of the 8341 participants who registered for an OPC engaged in a mindfulness session at least once during an OPC. A greater proportion of participants accessed only live sessions compared with those who accessed only recorded sessions (Table 2).

**Table 2:** Platform usage by completer status.

Usage metric		Did not complete both assessments (n=5934)	Completed pre and post PSS-4^a^ (n=2407)	Total (N=8341)	*P* value	
**Days (out of 30) used eMindful platform**						<.001
	N	5934	2407	8341		
	Mean (SD)	3.0 (5.6)	14.7 (10.7)	6.3 (9.1)		
	Median (range)	1.0 (0.0-30.0)	14.0 (0.0-30.0)	1.0 (0.0-30.0)		
**Number of live programs**						<.001
	N	5934	2407	8341		
	Mean (SD)	2.7 (6.0)	14.9 (14.8)	6.2 (10.9)		
	Median (range)	0.0 (0.0-127.0)	12.0 (0.0-151.0)	1.0 (0.0-151.0)		
**Days (out of 30) of live programs**						<.001
	N	5934	2407	8341		
	Mean (SD)	2.2 (4.6)	11.9 (10.2)	5.0 (8.0)		
	Median (range)	0.0 (0.0-30.0)	10.0 (0.0-30.0)	1.0 (0.0-30.0)		
**Number of recorded programs**						<.001
	N	5934	2407	8341		
	Mean (SD)	1.4 (4.8)	5.1 (10.9)	2.5 (7.3)		
	Median (range)	0.0 (0.0-139.0)	0.0 (0.0-122.0)	0.0 (0.0-139.0)		
**Days (out of 30) of recorded programs**						<.001
	N	5934	2407	8341		
	Mean (SD)	0.9 (3.1)	3.4 (7.0)	1.7 (4.7)		
	Median	0.0 (0.0-30.0)	0.0 (0.0-30.0)	0.0 (0.0-30.0)		
**Any eMindful use at all, n (%)**						<.001
	No	2588 (43.61)	246 (10.22)	2834 (33.98)		
	Yes	3346 (56.39)	2161 (89.78)	5507 (66.02)		
**Only used live programs, n (%)**						<.001
	No	4112 (69.30)	1411 (58.62)	5523 (66.22)		
	Yes	1822 (30.70)	996 (41.38)	2818 (33.78)		
**Only used recorded programs, n (%)**						<.001
	No	5345 (90.07)	2289 (95.10)	7634 (91.52)		
	Yes	589 (9.93)	118 (4.90)	707 (8.48)		
**Used both live and recorded programs, n (%)**						<.001
	No	4999 (84.24)	1360 (56.50)	6359 (76.24)		
	Yes	935 (15.76)	1047 (43.50)	1982 (23.76)		

^a^PSS-4: Perceived Stress Scale-4.

#### Missing Data

As shown in Table 3, 92.49% (n=7715) of the 8341 registered participants completed stress assessments prior to the OPC, whereas 30.09% (n=2510) completed the postassessment. Approximately one-quarter of the total sample (28.86%, n=2407) provided assessments both before and after their participation in the OPC. Most of these completers engaged in the platform at least once (89.78%, n=2161). Internal reliability on item-level data of the PSS-4 was high (Cronbach α=.82). Table 3 also presents perceived stress by completers and noncompleters.

**Table 3:** Perceived stress by completer status and time points.

Stress assessment			Did not complete both assessments (n=5934)		Completed both the pre and post PSS-4^a^ (n=2407)		Total (N=8341)		*P* value	
**Preprogram stress level**										<.001
	N	5308		2407		7715				
	Mean (SD)	6.0 (3.1)		5.6 (3.0)		5.9 (3.1)				
	Median (range)	6.0 (0.0 to 16.0)		5.0 (0.0 to 16.0)		6.0 (0.0 to 16.0)				
**Postprogram stress level**										.002
	N	103		2407		2510				
	Mean (SD)	5.1 (2.5)		4.3 (2.7)		4.3 (2.7)				
	Median (range)	5.0 (0.0 to 11.0)		4.0 (0.0 to 16.0)		4.0 (0.0 to 16.0)				
**Stress reduction (difference from post to pre; negative value indicates stress reduction)**										NA^b^
	N	0		2407		2407				
	Mean (SD)	NA		–1.3 (2.5)		–1.3 (2.5)				
	Median (range)	NA		–1.0 (–11.0 to –9.0)		–1.0 (–11.0 to –9.0)				

^a^PSS-4: Perceived Stress Scale-4.

^b^NA: not applicable.

### Engagement in Live and Recorded Training

**Figure 1:**
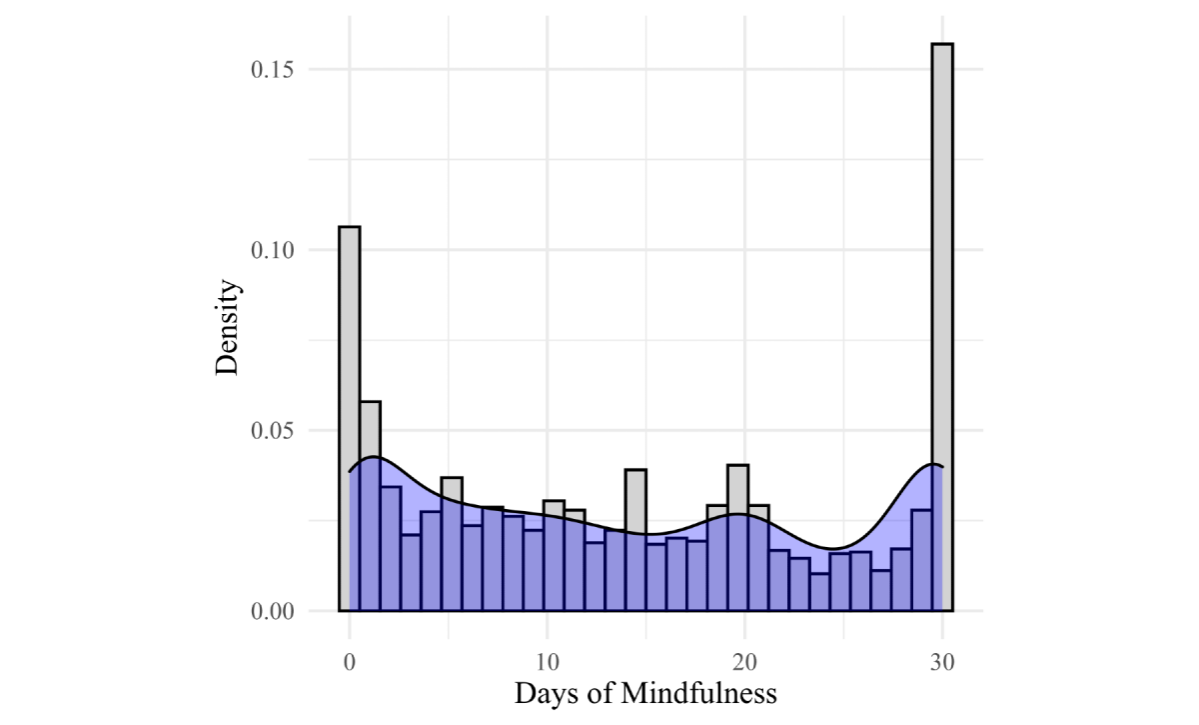
Days of mindfulness practice among completers.

**Figure 2:**
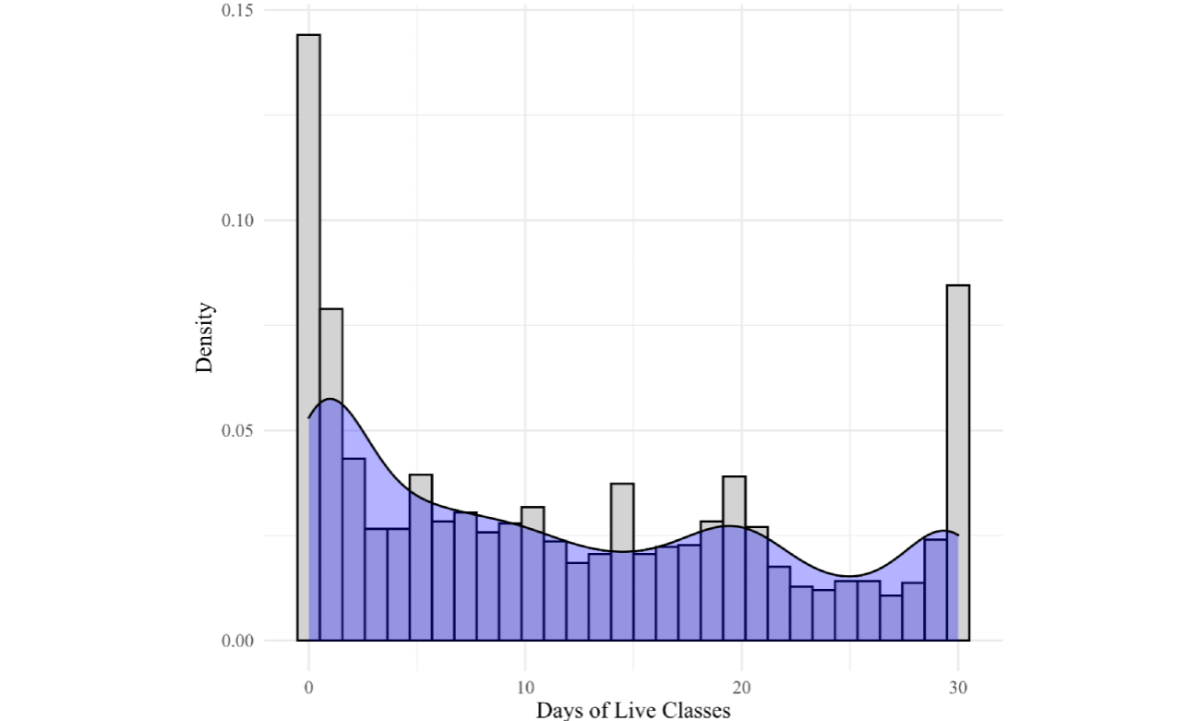
Completer engagement in live programs.

Figure 1 shows that approximately 15% of completers engaged every day and approximately 10% did not engage at all. Above approximately 3 days of engagement, the rates of engagement leveled off with some minor spikes at around 15 and 20 days of engagement. Figure 2 shows a similar picture in the engagement pattern for those using only live programs.

**Figure 3:**
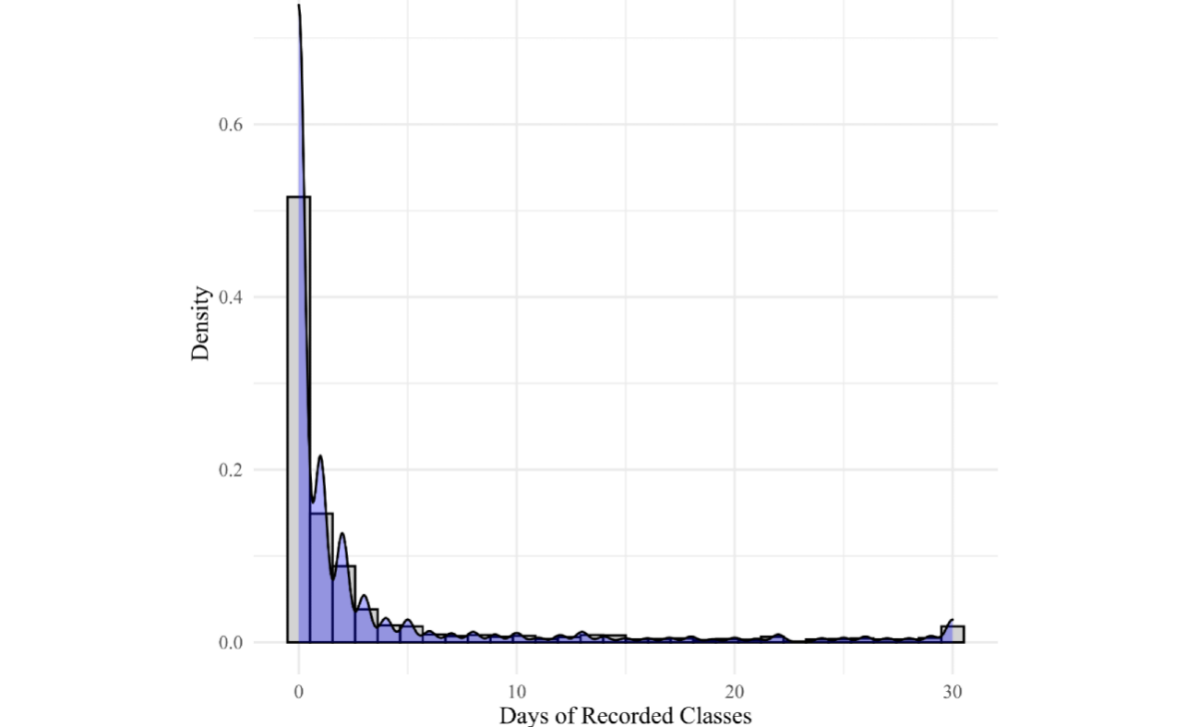
Completer engagement in recorded programs.

Use of recorded sessions presents a different story. As shown in Figure 3, many participants did not engage with recorded sessions at all (0 days: n=5652/8341, 67.76%), and for those who did, there was a steep decline of usage across the days. Most of those who used recordings at all (2689/8341, 32.22%) tended to use them 3-4 times at most. The remaining number of days using recordings was evenly scattered with a spike at 30 days (n=53 users).

### Sampling Bias

We constructed a linear mixed-effects model predicting the PSS-4 score and examining completer status at baseline, allowing for random intercepts of PSS-4 items within person. Those who completed both pre- and post-OPC assessments showed a tendency to have lower stress scores at baseline measurement (B=–0.89, SE=0.02, t_10,134.13_=–5.09; *P*<.001). This was a small but significant effect (Cohen *d*=–0.10). Given this potential source of bias, we included completer status as a control variable in all following analyses.

### Change in Stress Levels

**Figure 4:**
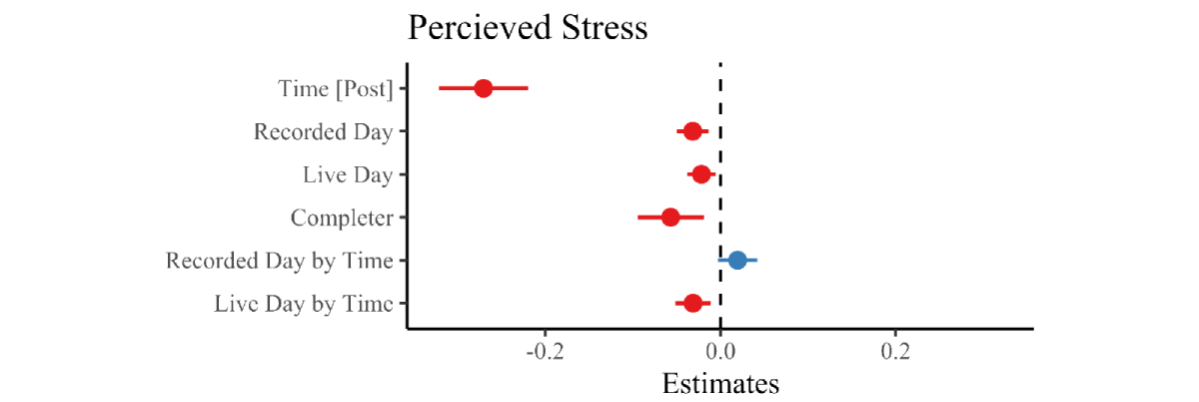
Linear mixed effects model of One Percent Challenge (OPC) usage controlling for completer bias.

We next sought to examine the degree of change in stress from pre- to post-OPC. We built upon the previous model’s random-effects structure by allowing for random slopes by time of measurement (pre- or post-OPC). We added time as a fixed effect and included completer status as a control variable in the model. We found a medium pre-post difference in stress levels (B=–0.32, SE=0.01, t_2393.25_=–24.99; *P*<.001, *d*=–1.02). The estimates are summarized in Table 4 and Figure 4, and detailed model statistics are shown in Table 5.

**Table 4:** Fixed effects and their interactions on stress in the mixed-effects linear models.

Predictors	Model 1		Model 2			Model 3	
	Estimates, B (95% CI)	*P* value	Estimates, B (95% CI)	*P* value	Estimates, B (95% CI)		*P* value
Intercept	1.50 (1.48 to 1.52)	<.001	1.53 (1.50 to 1.55)	<.001	1.53 (1.50 to 1.55)		<.001
Time [Post]	–0.32 (–0.35 to –0.30)	<.001	–0.25 (–0.31 to –0.20)	<.001	–0.27 (–0.32 to –0.22)		<.001
Completer status	–0.10 (–0.13 to –0.06)	<.001	–0.06 (–0.10 to –0.02)	.002	–0.06 (–0.09 to –0.02)		.003
Days of mindfulness (DoM)	—^a^	—	–0.03 (–0.04 to –0.01)	<.001	—		—
Time×DoM	—	—	–0.03 (–0.05 to –0.01)	.007	—		—
Log of recorded days	—	—	—	—	–0.03 (–0.05 to –0.01)		.001
Log of live teaching days	—	—	—	—	–0.02 (–0.04 to –0.01)		.007
Time×log of recorded days	—	—	—	—	0.02 (0.00 to –0.04)		.09
Time×log of live teaching days	—	—	—	—	–0.03 (–0.05 to –0.01)		.002

^a^Not included in model.

**Table 5:** Random effects on stress in the mixed-effects linear models.

Model statistic		Model 1	Model 2	Model 3
Variance (σ^2^)		0.43	0.43	0.43
**τ_00_**				
	Question: (Time:ID)	0.07	0.07	0.07
	Time:ID	0.07	0.07	0.07
	ID	0.36	0.36	0.36
Intracorrelation coefficient		0.54	0.54	0.54
**N**				
	Question	4	4	4
	Time	2_Time_	2_Time_	2
	ID	7281	7281	7281
Observations		40,984	40,984	40,984
Marginal *R*^2^/conditional *R*^2^		0.030/0.550	0.033/0.551	0.033/0.551

### Impact of Days of Mindfulness on Stress Reduction

Days of mindfulness was highly positively skewed (skew=1.50; D’Augustino test for skewness, *z*=41.69; *P*<.001). Thus, we proceeded with a log-transformed version of the days of mindfulness variable and its two contributors, days of live sessions and days of recorded sessions, for all following analyses. We added days of mindfulness and the interaction of time point and days of mindfulness to the model to further explore its potential moderating impact. This model explained significantly more variance over and above the time point alone (*χ^2^*_2_=27.98, *P*<.001). In addition, this log-transformed model explained the data better than the model that left days of mindfulness untransformed (*χ^2^*_0_=7.59, *P*<.001).

Days of mindfulness had an impact on the reduction of stress over and above the general effects of engaging in the OPC (B=–0.03, SE=0.01, *t*_3277.22_=–2.69; *P*=.007, *d*=–0.09). This represents an approximate 2.9% decrease in stress per day of mindfulness practice. Simply engaging in the OPC in some form over the time period of the OPC reliably reduced stress, and engaging over more days resulted in greater improvement. There was also some evidence that, accounting for all of these effects, individuals who were relatively less stressed overall were more likely to engage in more days of mindfulness (B=–0.03, SE=0.01, *t*_12,278.50_=–3.49; *P*<.001, *d*=–0.06; see Table 4).

### Relative Impact of Live Versus Recorded Programs on Stress Reduction

Breaking down days of mindfulness into days of live programs and days of recorded programs increased the explanatory power of the model (*χ^2^*_2_=9.06, *P*=.01). Participation in live programs drove decreases in stress (B=–0.03, SE=0.01, *t*_3258.61_=–3.03; *P*=.002, *d*=–0.11), whereas participation in recorded classes did not (B=–0.02, SE=0.01, *t*_3908.17_=1.79; *P*=.07, *d*=0.06; see Table 4). Participating in a live class resulted in a 3.1% reduction in stress.

### Impact of Participant Demographics

To examine the additional impact of participant demographics, we removed those who reported that they would “Prefer not to respond” in response to gender (n=120) or race (n=544). We then removed a cluster of participants whose age was listed as “99” (n=24). This was notably above the next maximum reported age of 79; therefore, these responses were deemed spurious. Given the proportions in the sample, race was coded as anyone selecting “white” alone and those who selected anything else, including other options among “white.” We pretested each demographic variable for differences in completer status to determine if there were additional sampling biases (see Table 6). Of these, women were marginally more likely to complete both time points of measurement (*P*=.09), and therefore we included the interaction of gender and completer status in the model to be conservative.

As shown in Table 6, we added the demographic variables to our previous model to evaluate their impact at baseline and their influence on change in stress over and above completer status and days of participating in live sessions or recorded sessions. Older participants had less stress at baseline (0.9% decrease in stress per year of age), with no clear difference in change in stress by age. Nonwhite participants had more stress at baseline (approximately 20.6% higher), but experienced greater reductions in stress from pre- to post-OPC (approximate 15.3% greater reduction) such that they had similar stress levels to those of the white participants post-OPC (who themselves experienced gains). The model statistics are summarized in Table 7.

**Table 6:** Demographic predictors of change in stress.

Predictors	Estimates, B (SE)	95% CI	*P* value (*df*=6893)
Intercept	1.91 (0.13)	1.66 to 2.17	<.001
Time [post]	–0.33 (0.11)	–0.56 to –0.11	.003
Completer status	–0.02 (0.05)	–0.12 to 0.07	.60
Gender [male]	0.18 (0.12)	–0.05 to 0.42	.12
Log of recorded days	–0.05 (0.02)	–0.08 to –0.02	.002
Log of live teaching days	0.00 (0.02)	–0.03 to 0.04	.86
Age	–0.01 (0.00)	–0.01 to 0.00	<.001
Race: Nonwhite	0.19 (0.06)	0.07 to 0.30	.001
Completer×Gender [male]	–0.25 (0.12)	–0.48 to –0.02	.03
Time×Log of recorded days	0.00 (0.02)	–0.03 to 0.03	.99
Time×Log of live teaching days	–0.06 (0.02)	–0.09 to –0.02	.006
Time×Gender [male]	–0.01 (0.05)	–0.11 to 0.10	.87
Time×Age	0.00 (0.00)	0.00 to 0.01	.06
Time×Nonwhite	–0.14 (0.05)	–0.24 to –0.04	.006

**Table 7:** Random effects of the model including demographic factors to predict changes in stress levels.

Model statistic		Value
σ^2^		0.42
**τ_00_**		
	Question:(Time:ID)	0.08
	Time:ID	0.05
	ID	0.27
Intracorrelation coefficient		0.48
**N**		
	Question	4
	Time	2
	ID	703
Observations		6911
Marginal *R*^2^/conditional *R*^2^		0.050/0.509

## Discussion

### Principal Findings

This study is the first to explore the relative contribution of live and recorded online mindfulness training to the reduction of stress. In this study of participants in the eMindful OPC, completers demonstrated a reduction in stress across their 30 days of participation with a medium effect size per Cohen *d* (95% CI 0.54-0.63). This is consistent with multiple meta-analyses of RCTs examining the impact of online mindfulness programs on perceived stress [3,29]. Perhaps more importantly, this is the first paper to report that participants’ use of live teaching clearly drives the effects on stress reduction.

First, participating *at all* in eMindful OPC services is beneficial from a stress reduction perspective. Moreover, those who used live teaching and supplemented with recordings demonstrated the greatest effects, with an average improvement of 1.23 points on the PSS-4. Similarly, participating in only live teaching during the OPC conferred a smaller, but significant, reduction in stress. Although using only recorded programs or “on demand” content for practice did not reduce stress, the use of recordings did serve an important function. On average, participants used recordings only a handful of times across the month, suggesting that this minor usage is sufficient to supplement a regular daily mindfulness practice. Notably, running this same model using pure attendance numbers rather than days of practice did not show effects, indicating that changes in stress are more related to regular daily practice than the number of practices completed.

Second, in addition to benefits occurring from participating *at all* in the OPC, our data confirm that the more days one practices, the better. Stress reduction occurred in our sample from participating at all, and the remaining distance toward enduring change occurred at a rate of approximately 2.9% reduction per day of practice.

### Why Might Live Online Teaching Drive Stress Reduction?

It makes intuitive sense that live programs would drive stress reduction, as the social component of live teaching, group discussion, and the importance of processing personal experiences are all critical elements in adult learning, particularly for higher-order thinking skills [2]. To sort through the high volume, rapidly changing, and often contradictory information coming at employees on a daily basis, higher-order thinking skills are imperative. In fact, such skills are thought to better prepare individuals to manage change than any knowledge or skill set: “The need to provide a highly educated, skilled workforce capable of providing solutions to 21st century challenges and issues has never been greater” [36]. The education literature can provide a better understanding of this phenomenon.

In academia, studies of online learning have mostly focused on learning through asynchronous platforms where participants access resources such as recorded lectures, readings, quizzes, and discussion boards at their convenience [37]. With the rapidly evolving technology of webcams and microphones to allow real-time participation, synchronous online learning has been increasing. Although there are still few studies on the topic, the research performed to date shows that participants indicate that any possible frustration with the technology itself is overridden by the convenience of synchronous online learning [37]. This literature will likely evolve rapidly given the move from offline to online education in a short period during the COVID-19 pandemic [38], which will hopefully provide even greater insight into online learning.

The peer-reviewed literature on the effectiveness of online learning to date can help explain why the live teaching component is the driver of our outcomes. Researchers of online learning have long supported the value of social interaction as a crucial element in the learning process [39]. Despite some equivocal evidence, successful synchronous courses provide opportunities for participants to share experiences and interact with others. This opportunity is not available in recorded programs or on static apps. Live teaching allows for peer-to-peer as well as peer-to-instructor interaction, a crucial element in the learning process. Research shows that peer-to-instructor interaction remains strong in online synchronous learning, sometimes superior to in-person learning, and that there is greater variability regarding peer-to-peer interaction [37]. The quality of peer-to-peer interaction depends in part on the program’s use of polling and chats in addition to microphones that allow participants to express their thoughts and experiences. Learning theory clarifies that adults use personal life experiences as a framework for all subsequent learning [40]. Processing their mindfulness experiences together in class provides opportunities for participants to connect to their current learning. This cognitive scaffolding is crucial for higher-order thinking and occurs through interaction.

Only synchronous online programs allow for discussion with others in the class as well as with the instructor. Since participants are processing their own experiences in real time, each comment can shift the direction of thinking in another person. Owing to this constant shifting, individuals tend to reach higher levels of thinking as they respond to each other’s statements [39]. Discussion and group input thus invite individuals to reach higher levels of thinking, which may be important in navigating the constant change and uncertainty required in today’s world.

The education literature can provide more information regarding adult learners. There is also a wide range of participant confidence in contributing online, with some feeling less confident to contribute online versus in person and others expressing more confidence to contribute online [37]. Whether use of the online synchronous classroom tends to engender more or less confidence in participating may depend on the level of interactive opportunities the program provides. When there is reduced interaction online versus in person, a likely explanation has to do with how technology is used within sessions. The technology has developed to allow for considerable peer-to-peer interaction, with features such as polls, surveys, and breakout rooms; however, in practice, the interactive technology is not always used to its full potential. Ng and Jeffery [41] found that teachers using new software tended to stick to the traditional didactic lecture style of teaching, estimating that they devoted a quarter of the time to interactive activities online than they would in a comparable classroom. Fortunately, the competitive nature of the corporate world has demanded creative methods of engagement from onboarding through delivery of each session. OPC sessions employ a small didactic nugget with inquiry that invites participants to place the lesson in daily context. Most of the session time is focused on experiential learning, community practice, and processing the experience.

Observations from the industrial organizational literature also support the importance of a live teacher. In studies on performance feedback, researchers note the importance of learners perceiving “social presence,” a sense of human warmth and being with another person [42]. In addition to influencing the perceived utility of feedback systems in learning, social presence appears to be particularly important in distance-based learning [42]. Not surprisingly, human feedback is perceived as having greater warmth and sense of closeness than machine-generated feedback. Similarly, the richer the feedback media (eg, containing visual and auditory input), the greater the perceived social presence as it contains greater social cues [42]. Similar observations have been demonstrated in the behavioral change literature. For example, both human-generated email feedback and computer-tailored feedback to participants in a weight loss trial appeared to have the same impact at 3 months, but only the improvement from the human-generated email group was maintained at 6 months [43,44]. The use of recorded practices and computer-tailored models is likely to be quite nuanced. It is clear that multiple variables impact behavior change and learning outcomes from online participants, such as the education of participants [44]. The education literature also shows the potential impact of age on one’s preference for live online versus recorded teaching. Depending on the context, student preferences for recorded training versus live online training have been noted, particularly for older students in situations of content learning [45]. Our results did not show age as a moderator of our finding that the live programs, supplemented by on-demand sessions, is what drove the reduction in stress.

Although eMindful’s focus has been live teaching through a virtual classroom, the company developed an app and recorded options for daily use on the platform to further support practice. Our findings suggest that participants obtained the most benefit when using the on-demand options as a supplement to support live practice. This effect has been detected in at least one other trial as well. In an RCT comparing participants with access to daily guided meditations to participants who had the same access but also received a 1-hour web-based live training session for 6 weeks, Wahbeh and Oken [46] showed that usage of the live web platform increased daily meditation practice.

### Limitations of the Study

This study has several limitations that should be considered in weighing the findings. First, the sample for this study came from a large pool of participants in a particular program (the OPC) at a single mindfulness training company. Hence, it is unclear if the findings would generalize to other programs in the company or to other companies. Second, there is a considerable amount of missing data in the sample. Analyzing data from corporate programs is inherently challenging, since each corporate client dictates the data to be collected, providing considerable nonrandom variability. For example, missingness for sociodemographic data is a result of when the participants enrolled, the processes at eMindful at the time, and each organization’s preferences. Hence, the demographics provided cannot be assumed to be a random representation of the entire database, or of corporate employees in general. Similarly, missingness for outcomes data appears to be nonrandom, making it extremely important that the analyses allowed for potential sources of bias. Fortunately, our analyses did take potential sources of bias into consideration. Completers showed a 0.045-point lower pre-OPC stress level compared with that of noncompleters. This completion bias suggests that noncompleters were slightly more stressed. Based on these data, we would expect that if these noncompleters did complete both pre- and post-OPC measures, the effects observed on outcomes would only increase. However, this may depend on engaging them more in services. Per these analyses, if all people who engaged in services completed both surveys, we may reasonably assume that the models presented regarding the reduction of stress are conservative estimates of the potential of the OPC to reduce stress. Finally, the baseline rates of using recorded sessions were much lower than those of using live sessions. We do not know what impact this had on our results. We attempted to account for this bias by analyzing log-transformed variables and including the effect of engaging in recorded sessions at all. Although our findings may speak to ecologically valid situations, the field would benefit from additional study of well-controlled comparisons of recorded versus live sessions that compel equal dosage in a randomized fashion.

### Conclusion

Participation *at all* with the eMindful OPC reduced stress, and live online teaching drove this outcome. Regular practice across the month led to an even greater reduction in stress. The use of recorded or on-demand offerings to supplement live practices conferred the strongest likelihood of achieving a clinically significant change in stress levels. Our findings are in stark contrast to the rapid evolution of online mindfulness training for the workplace. Specifically, the market is reproducing apps and recorded teaching at an unprecedented pace, whereas our results demonstrate that live teaching leads to greater stress reduction.

## Abbreviations

OPC: One Percent Challenge
PSS-4: Perceived Stress Scale-4
RCT: randomized controlled trial
